# Limitations of free-form-text diagnostic requisitions as a tool for evaluating adherence to appropriate use criteria for transthoracic echocardiography

**DOI:** 10.1186/1476-7120-13-4

**Published:** 2015-01-15

**Authors:** Behnam Banihashemi, Kasra Maftoon, Benjamin J W Chow, Jordan Bernick, George A Wells, Ian G Burwash

**Affiliations:** Department of Medicine, Division of Cardiology, University of Ottawa Heart Institute, University of Ottawa, 40 Ruskin Street, Rm 3407B, K1Y 4W7 Ottawa, Ontario Canada

**Keywords:** Transthoracic echocardiography, Appropriate use criteria, Diagnostic requisitions

## Abstract

**Background:**

Monitoring the adherence to Appropriateness Use Criteria (AUC) has been identified as an important component for the accreditation of echocardiography laboratories. Referral requisitions are a logical tool to rapidly determine the appropriateness of transthoracic echocardiography (TTE) referrals, however data is lacking. We investigated whether standard free-form-text TTE referral requisitions can be used to evaluate AUC adherence.

**Methods:**

Consecutive TTE referral requisitions to the University of Ottawa Heart Institute echocardiography laboratory were reviewed over a four-week period. Indication on the requisition was matched with the relevant indication on the 2011 American College of Cardiology Foundation (ACCF) AUC. Requisitions that did not provide sufficient information to identify the relevant AUC indication were identified as inadequate. For inadequate requisitions, reason for the referral was clarified through medical records and referring physicians.

**Results:**

Of the 1303 requisitions, 26.2% did not provide adequate information to determine adherence to AUC, despite a non-adherence (inappropriate) rate of only 6.1% in the referral population. Indication for referral, physician specialty, outpatient status, and prior echocardiogram were independent predictors of inadequate requisitions (p < 0.001, respectively). The most common reasons for inadequate requisitions were a failure to report: 1) change in clinical status, 2) date of a prior echocardiogram, and 3) type and/or severity of a valve lesion. Inclusion of this information would have decreased the inadequacy rate by 56%.

**Conclusion:**

In a large, academic echocardiography laboratory, over one quarter of free-form-text TTE requisitions are inadequate to evaluate AUC adherence. Structured requisition formats requiring AUC-relevant information are needed to facilitate the practical application of AUC in the echocardiography laboratory.

**Electronic supplementary material:**

The online version of this article (doi:10.1186/1476-7120-13-4) contains supplementary material, which is available to authorized users.

## Background

Utilization of echocardiography has grown rapidly in recent years [1, 2], and exceeds the increase in cardiovascular disease prevalence [3]. The associated escalating health care costs have led health care insurers to question whether the current use of echocardiography is appropriate and guided by best practice guidelines [4, 5]. To facilitate the rationale use of imaging services, The American College of Cardiology Foundation (ACCF) Task Force, in conjunction with key specialty and subspecialty societies, developed guidelines for the appropriate use of cardiac diagnostic services, and similar processes are now under development in other countries [6–11]. The first set of Appropriate Use Criteria (AUC) for echocardiography were published in 2007 and updated in 2011 [8, 9]. While physicians are expected to use these criteria to guide their management decisions, the onus for the application of AUC has in part become the responsibility of the echocardiography laboratory. Demonstration of adherence to AUC is now required for accreditation of echocardiography laboratories by government insurers and international organizations, such as the Intersocietal Commission for the Accreditation of Echocardiography Laboratories (ICAEL) [12, 13].

The diagnostic requisition completed by the referring physician is the logical tool for echocardiography laboratories to evaluate AUC adherence in real-time. However, there is no data on the use of the diagnostic requisition for this purpose. The vast majority of echocardiography laboratories employ free-form-text requisitions in either paper or electronic format, relying on the referring physician to provide the indication for the referral and relevant supporting clinical information. However, referring physicians come from diverse clinical specialties and may fail to provide the information required for an echocardiography laboratory to evaluate the appropriateness of the referral. The objective of this study was to determine if free-form-text echocardiography requisitions completed by the referring physician can be used to evaluate AUC adherence for transthoracic echocardiography (TTE), and additionally, to identify potential barriers to this process.

## Methods

### Medical center and study population

The University of Ottawa Heart Institute (UOHI) is a large academic cardiovascular center within The Ottawa Hospital in Ottawa, Ontario, Canada, and provides cardiology care to 1.5 million residents of Ottawa and Northeastern Ontario. The UOHI echocardiography laboratory is staffed by four geographic full-time Level 3 echocardiographers [14], and provides outpatient and inpatient echocardiography services for both community and hospital based primary care physicians and specialists. The laboratory is accredited by ICAEL and has an annual volume of 19092 echocardiography studies (2013). The study population consisted of 1303 consecutive patients who were referred for TTE to the echocardiography laboratory at the UOHI over a 4-week period in August.

## Echocardiography requisition review protocol

The TTE referral requisition completed by the referring physician was prospectively reviewed for the study indication prior to completion of the echocardiogram (Additional file 1). Using information extracted from the requisition, the study indication was matched with the relevant indication on the ACCF 2011 AUC and reported as appropriate, uncertain, inappropriate or unclassified [9]. Identification of the relevant indication on the ACCF 2011 AUC was made by the consensus of two reviewers, and a third reviewer, if consensus was not achieved. Requisitions that did not provide sufficient information to identify the relevant ACCF 2011 AUC indication and appropriateness classification were recorded as inadequate. The reason for inadequacy of the requisition was documented by identifying the “missing” information that if provided would have allowed determination of the relevant AUC indication (failure to report on a change in clinical status [change in symptoms or signs], inadequate clinical information excluding change in clinical status, failure to provide the date of a previous echocardiogram, failure to report the type and/or severity of a valve lesion, and failure to report on the status of a congenital defect). For inadequate requisitions, the computerized echocardiography database and electronic medical records were subsequently searched and the referring physician contacted to clarify the reason for the study.

Patient age, gender, inpatient or outpatient status, previous echocardiography examination and referring physician specialty (primary care physician [family or emergency medicine], cardiologist, internal medicine specialist [excluding cardiology], cardiac surgeon, all other specialists) were recorded.

The institutional research ethics board approved the study.

### Statistical analysis

Continuous variables are reported as medians with interquartile ranges; and categorical variables, as percentages. Comparisons were made using *χ*2 tests for categorical variables. To determine independent predictors of inadequate requisitions, a multivariable logistic regression analysis was performed. Each model was adjusted for patient factors thought to be potentially associated with inadequate requisitions. These candidate explanatory variables included inpatient or outpatient status, previous echocardiography examination, referring physician specialty, indication for study as categorized by the ACCF 2011 AUC [9], age and gender. Model discrimination and calibration were assessed using the c-statistic and Hosmer-Lemeshow goodness-of-fit test, respectively.

## Results

The demographics of the 1303 patients, including indication for TTE referral, referring physician specialty, and outpatient/inpatient status are shown in Table 1. The study population was primarily outpatient based with cardiologists accounting for the largest referring physician specialty. There was a wide distribution of study indications with 86% of referrals included in the ACCF 2011 AUC categories of 1) general evaluation of cardiac structure and function, 2) evaluation of valvular function, 3) evaluation of intracardiac and extracardiac structures and chambers, and 4) evaluation of hypertension, heart failure, or cardiomyopathy (Tables 1 and 2). The most common specific study indication for each referring physician group is shown in Table 3.

**Table 1:** Demographics and characteristics of the study population

Variable	Study population
	n = 1303 (%)
Age*	65 (52-76)
Male	731 (56.1)
Inpatients	318 (24.4)
Referring physician	
Primary care physicians	252 (19.3)
Cardiologists	639 (49.0)
Internal medicine [excluding cardiology]	136 (10.4)
Cardiac surgeons	85 (6.5)
Other specialists	191 (14.7)
Prior transthoracic echocardiogram	712 (54.6)
Inadequate requisition	341 (26.2)
Indication^†^	
General evaluation of cardiac structure and function	343 (26.3)
Evaluation of valvular function	315 (24.2)
Evaluation of intracardiac and extracardiac structures and chambers	206 (15.8)
Evaluation of hypertension, heart failure, or cardiomyopathy	202 (15.5)
Cardiovascular evaluation in an acute setting	106 (8.1)
Adult congenital heart disease	49 (3.8)
Evaluation of aortic disease	24 (1.8)

*Data are presented as medians (25th-75th percentile).

^†^Based on the ACCF 2011 AUC categories of indications [9].

**Table 2:** Most common specific indications for transthoracic echocardiography*

AUC indication no.	Description	Number of requisitions (% of population)
58	Suspected cardiovascular source of embolus	162 (12.4)
1	Symptoms or conditions potentially related to suspected cardiac etiology	134 (10.3)
34	Initial evaluation when there is a reasonable suspicion of valvular or structural heart disease	72 (5.5)
5	Sustained or non-sustained atrial fibrillation, SVT, or VT	64 (4.9)
37	Re-evaluation of valvular heart disease with a change in clinical status or cardiac exam or to guide therapy	64 (4.9)
24	Initial evaluation of ventricular function following ACS	53 (4.1)
70	Initial evaluation of known or suspected HF (systolic or diastolic) based on symptoms, signs, or abnormal test results	51 (3.9)
2	Prior testing that is concerning for heart disease or structural abnormality	42 (3.2)

*Specific indication number and associated descriptions are based on the ACCF 2011 AUC for Echocardiography [9].

[AUC = appropriate use criteria].

**Table 3:** Most common specific indications for transthoracic echocardiography by referring physician specialty*

Referring physician	Specific indication (AUC indication no.)	Number of requisitions (% of specialty population)
Primary care physicians	Symptoms or conditions potentially related to suspected cardiac etiology (1)	59 (23.4)
Internal Medicine [excluding cardiology]	Evaluation of suspected pulmonary hypertension (15)	17 (12.5)
Cardiologists	Symptoms or conditions potentially related to suspected cardiac etiology (1)	58 (9.1)
Cardiac surgeons	Initial postoperative evaluation of prosthetic valve for establishment of baseline (47)	22 (25.9)
Other specialists	Suspected cardiovascular source of embolus (58)	134 (70.2)

*Specific indication number and associated descriptions are based on the ACCF 2011 AUC for Echocardiography [9].

[AUC = appropriate use criteria].

**Figure 1:**
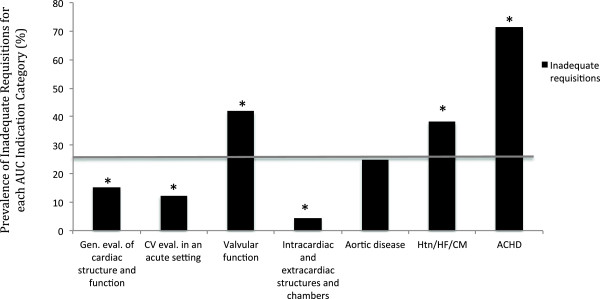
**Prevalence of inadequate requisitions by ACCF 2011 AUC indication category.** [Data are presented as a percentage of each indication category. The grey line depicts the prevalence in the overall population. *represents p < 0.001 when the prevalence in the individual subgroups are compared to that of the overall population]. ACHD, Adult Congenital Heart Disease; CM, Cardiomyopathy; CV, Cardiovascular; Gen. Eval., General Evaluation; HF, Heart Failure; Htn, Hypertension.

**Figure 2:**
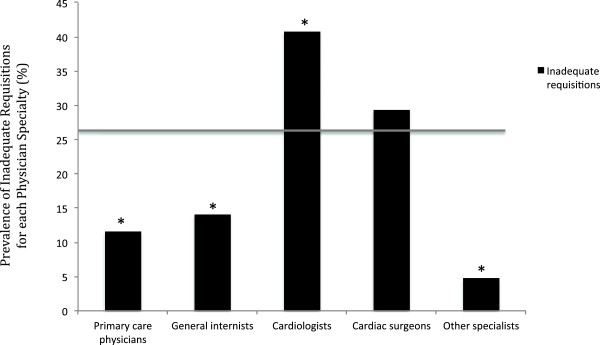
**Prevalence of inadequate requisitions by referring physician specialty.** [Data are presented as a percentage for each specialty. The grey line depicts the prevalence in the overall population. * represents p < 0.001 when the prevalence in the individual subgroups are compared to that of the overall population].

**Figure 3:**
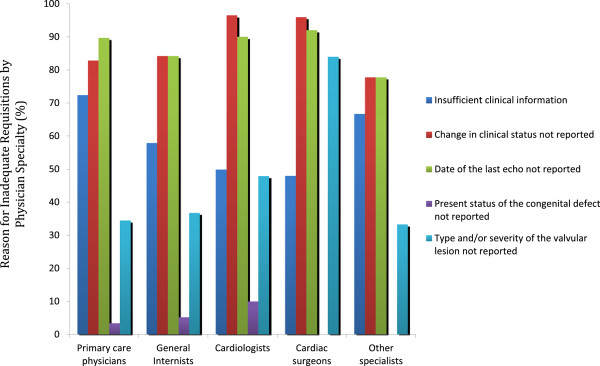
**Reasons for inadequate requisitions by referring physician specialty.** [Data are presented as a percentage of inadequate requisitions for each specialty].

Of the 1303 TTE referral requisitions, 26.2% (n = 341) provided inadequate information to evaluate the adherence to AUC (Table 4). The most common reasons for requisition inadequacy are shown in Table 4. The three most common reasons were failure of the referring physician to report: 1) a change in clinical status (n = 321), 2) the date of a previous echocardiogram (n = 305), and 3) the type and/or severity of a valve lesion (n = 165). Inclusion of this information on the referral requisition would have decreased the requisition inadequacy rate to 11.6% (56% reduction).The prevalence of an inadequate requisition in each ACCF 2011 AUC indication category is shown in Figure 1. Three of these categories were more likely to contain inadequate information on the requisition as compared to the overall referral population: 1) adult congenital heart disease (71.4% of requisitions inadequate), 2) evaluation of valvular function (41.9% of requisitions inadequate), and 3) evaluation of hypertension, heart failure or cardiomyopathy (38.1% of requisitions inadequate) (p < 0.001 for all 3 groups). Inadequate requisition information was present on 8.0% of in-patient referrals and 31.9% of outpatient referrals (p <0.001). Cardiologists were more likely than other referring physician specialties to provide inadequate information on the requisition (40.8% of requisitions; p < 0.001) (Figure 2). Although cardiologists accounted for 49.0% of TTE referrals, they accounted for 75.9% of inadequate requisitions. Primary care physicians and internal medicine specialists [excluding cardiology] were less likely to provide requisitions with inadequate information (11.5% and 14.0% respectively, p < 0.001 for both). The reasons for an inadequate requisition were consistent across referring physician specialties (Figure 3).

**Table 4:** Reason for inadequate information on echocardiography requisition*

Variable	Number of requisitions (%)
Failure to report on a change in clinical status	321 (94.1)
Date of last echo not reported	305 (89.4)
Failure to report on the type and/or severity of a valvular lesion	165 (48.4)
Inadequate clinical information on the requisition (excluding change in symptoms or signs)	135 (39.6)
Present status of congenital defect not reported	28 (8.2)

*Data are presented as absolute number of requisitions (percentage of inadequate requisitions).

**Figure 4:**
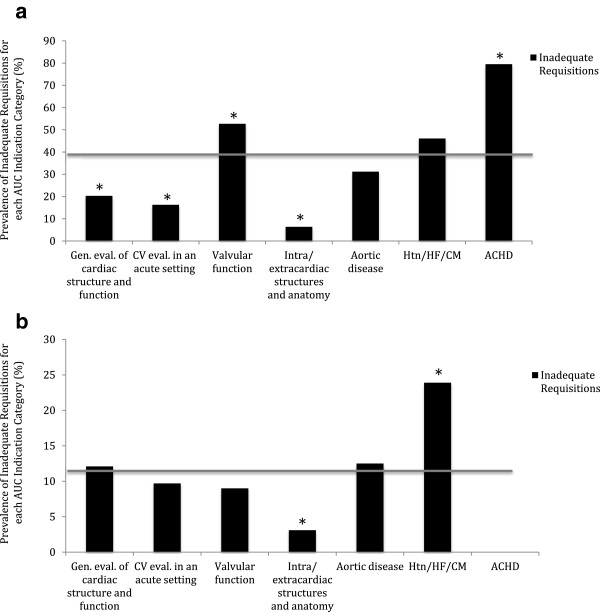
**Prevalence of inadequate requisitions by ACCF 2011 AUC indication category in (a) patients with a previous echocardiogram (n = 712) and (b) patients without a previous echocardiogram (n = 591).** [Data are presented as a percentage of each indication category. The grey line depicts the prevalence in each population. * represents p < 0.001 when the prevalence in the individual subgroups are compared to that of the overall population]. ACHD, Adult Congenital Heart Disease; CM, Cardiomyopathy; CV, Cardiovascular; Gen. Eval., General Evaluation; HF, Heart Failure; Htn, Hypertension.

Patients with a prior echocardiogram were more likely to have inadequate information on the requisition compared to patients with no prior echocardiogram (38.8 vs. 11.3%, p < 0.001). The prevalence of inadequate requisitions in each ACCF 2011 AUC indication category in patients with and without a prior echocardiogram is shown in Figure 4. In patients with a prior echocardiogram, two categories were more likely to contain inadequate information on the requisition: 1) adult congenital heart disease (79.6% of requisitions, p < 0.001), and 2) valvular function (52.7% of requisitions, p < 0.001). The category of evaluation of hypertension, heart failure or cardiomyopathy approached statistical significance (46.2% of requisitions, p = 0.059). In patients with no prior echocardiogram, only the category of evaluation of hypertension, heart failure or cardiomyopathy was more likely to contain inadequate information (23.9% of requisitions, p < 0.001).

On multivariate analysis, indication for referral, referring physician specialty, outpatient status, and prior echocardiogram were independent predictors of a requisition with inadequate information to evaluate adherence to AUC (Table 5). Cardiologists were 5.5 times [95% CI: 3.4-8.8] more likely than primary care physicians to provide inadequate information on the requisition. Patients with no prior echocardiogram and inpatients were less likely to have a requisition with inadequate information. The model c-statistic was 0.84 and P value for Hosmer-Lemeshow goodness-of-fit test was 0.12, demonstrating adequate discrimination and calibration, respectively.

**Table 5:** Independent predictors of an inadequate echocardiography requisition

Variable	Adjusted odds ratio (95% CI)	P value
Inpatient	0.20 (0.12-0.32)	<0.001
No previous echocardiogram	0.39 (0.27-0.56)	<0.001
Referring physician specialty		<0.001
Indication for study*		<0.001
Age > 65	0.89 (0.64-1.23)	0.54
Female gender	1.01 (0.74-1.38)	0.98

*Based on the ACCF 2011 AUC categories of indications [9].

After review of the referral requisitions, including the electronic records and contact with the referring physicians where necessary, only 6.1% (n = 79) of TTE referrals were classified as non-adherent (inappropriate) by AUC. The non-adherence rate was 2.0% (n = 19) in patients with an adequate requisition and 17.6% (n = 60) in patients with an inadequate requisition (p < 0.01).

## Discussion

The ACCF AUC has been rapidly adopted in the United States as a useful tool to guide the appropriate use of echocardiography [15–24], and AUC are being integrated into the delivery of echocardiography services in other countries [10–13]. Demonstration of AUC adherence by echocardiography laboratories is already required for accreditation by government and international organizations (ICAEL) and this trend is expected to extend to other jurisdictions [12, 13]. To date, published studies evaluating the appropriateness of echocardiography have employed an extensive review of the patient’s medical record, including diagnostic requisitions, written/electronic charts, electronic databases and contact with the referring physician [15–24]. This retrospective process requires a significant investment of time and personnel by an echocardiography laboratory, is difficult without an available electronic access to the patient’s complete medical record, is limited in scope by only sampling a portion of an echocardiography laboratory’s volume, and most importantly, does not allow for the “real-time” application and identification of AUC adherence for every examination. There is a need to make the process of evaluating and applying AUC in the echocardiography laboratory simple and practical to allow for the “real-time” identification of non-adherent and potentially inappropriate studies [25].

The echocardiography requisition completed by the referring physician is the logical tool for this process and most echocardiography laboratories use free-form-text diagnostic requisitions, in which the referring physician provides the indication for the referral and relevant supporting clinical information. Unfortunately, we have demonstrated that 26.2% of free-form-text referral requisitions to a large academic echocardiography laboratory do not provide enough information to determine AUC adherence, despite an AUC non-adherence (inappropriate) rate of only 6.1% in the referral population. Four factors were predictive of inadequate information on the referral requisition: 1) indication for referral, 2) referring physician specialty, 3) outpatient status, and 4) prior echocardiogram.

The vast majority of inadequate requisitions were related to the referring physician’s failure to 1) report on a change in clinical status, 2) provide the date of a previous echocardiogram, and 3) report the type and/or severity of a valve lesion. This information is usually available to the referring physician, and if included, would have reduced our rate of inadequate requisitions by 56%. We would recommend that all TTE referral requisitions contain mandatory fields which require referring physicians to provide this information.

Cardiologists were more likely than other referring physician specialties to provide inadequate information on the requisition (41% of all requisitions). This positive association persisted after correcting for multiple confounding variables. Multiple studies have shown that the adherence to AUC is greater by cardiologists compared to other physician specialties [15, 16], and factors other than appropriateness are relevant to the completion of an echocardiography requisition. Primary care or non-cardiology specialists may provide more information on the requisition because they may not be as certain as their cardiology colleagues about the diagnosis or relevance of clinical information, thereby allowing a more frequent determination of AUC adherence. In contrast, cardiologists may be less concerned about “justifying” their request for an echocardiogram as they know that the examination is “appropriate”. Nonetheless, non-adherent and potentially inappropriate referrals originate from all referring specialties and need to be identified to avoid potentially unwarranted healthcare costs and unnecessary delays to diagnosis and treatment for all patients requiring echocardiography. In this regard, free-form-text referral requisitions are inadequate for the monitoring and application of AUC adherence, especially among referrals from cardiologists, which accounted for almost half of our referral population.

Similarly, free-form-text referral requisitions originating from the outpatient setting appear particularly problematic for evaluating AUC adherence. Almost one third of outpatient requisitions were inadequate to determine AUC adherence, a significantly greater prevalence than observed for inpatient requisitions. Often inpatients have had a dramatic change in clinical status, which is documented on the requisition and justifies the examination. In contrast, referring physicians may fail to report more subtle changes in clinical status on an outpatient requisition that warrants the echocardiogram, despite the importance of this information to evaluate AUC adherence. In our center, inpatients are primarily cared for by medical trainees, who are often entrusted with the completion of requisitions. These less experienced physicians generally provide more clinical information on the requisition because they are not sure of the significance or potential relevance, thereby allowing a determination of AUC adherence. The limitation of the free-form-text referral requisition in outpatient referrals is compounded since outpatients account for the majority of most echocardiography laboratory volumes, and access to additional medical information to evaluate AUC adherence may not be easily available.

A prior echocardiogram was a strong predictor of an inadequate requisition and the prevalence of inadequate requisitions was particularly high in the AUC categories of adult congenital heart disease, valvular function and evaluation of hypertension, heart failure or cardiomyopathy. The vast majority of these patients were likely undergoing follow-up examinations for previously identified cardiac disease, and in this regard, detailed information on the disease severity (i.e. mild, moderate, or severe valve lesion), completeness of a previous repair, timing of a prior assessment and change in clinical status are required to identify the relevant ACCF 2011 AUC indication and appropriateness classification. This represents a significant challenge to free-form text requisitions as failure of a referring physician to include all this specific relevant information would result in an inadequate requisition.

Free-form-text referral requisitions, whether in paper or electronic format, are inherently limited as a tool for evaluating AUC adherence since they require referring physicians to both identify and document the important, relevant clinical information contained within the AUC guidelines. It is likely unreasonable to expect physicians from different specialties to be thoroughly versed in the specific components of each AUC indication and to transmit the essential information to a free-form-text referral requisition. In this regard, the adoption of structured electronic referral requisitions using a decision tree algorithm encompassing the specifics of the AUC guidelines could facilitate the process of evaluating and applying AUC in an echocardiography laboratory [25]. Non-adherent and potentially unnecessary studies could be identified at the time of requisition completion by either the referring physician or echocardiography laboratory and patient management modified if appropriate.

### Study limitations

Our study was conducted in an academic, tertiary care medical center echocardiography laboratory accessible to both community and hospital based primary care physicians and specialists. The UOHI echocardiography laboratory does not restrict referral access to specific physicians or patient populations and these relationships should be generalizable to similar institutions. However, our observed rate of inadequate referral requisitions might vary in an institution with a significantly different distribution of referral diagnoses, referring physician specialties and inpatient/outpatient ratios since these factors affect the rate of inadequate requisitions. Nevertheless, the large number of echocardiograms and referring physicians analyzed in this study has allowed us to identify the important limitations of free-form-text referral requisitions as a tool for the evaluation and application of AUC adherence.

There is no standard requisition for echocardiography and the results might differ in an echocardiography laboratory using a significantly different requisition format. However, the UOHI echocardiography laboratory requisition is similar in content to those widely used by echocardiography laboratories and contains all the elements recommended by accrediting echocardiography organizations such as ICAEL [12, 13].

The investigators were not blinded to the patient demographics or referring physician when reviewing the echocardiography requisition. While a process of consensus review was employed, we cannot exclude the possibility of the introduction of bias during determination of the adequacy of the echocardiography requisition, or the determination of appropriateness of the examination.

## Conclusion

In a large, consecutive population of patients referred to an academic echocardiography laboratory, free-form-text referral requisitions for TTE failed to provide adequate information to determine AUC adherence in 26% of referrals. Inclusion of information on 1) a change in clinical status, 2) the date of a previous echocardiogram, and 3) the type and/or severity of a valve lesion on the referral requisition could reduce the prevalence of inadequate TTE requisitions by more than 50%. Structured requisition formats that require referring physicians to provide AUC-relevant information are needed to facilitate the monitoring and application of AUC in the echocardiography laboratory.

## Electronic supplementary material

Additional file 1: **University of Ottawa Heart Institute Echocardiography Requisition.** (PNG 95 KB)

## Abbreviations

ACCF: American College of Cardiology Foundation
AUC: Appropriateness use criteria
ICAEL: Intersocietal Commission for the Accreditation of Echocardiography Laboratories
TTE: Transthoracic echocardiography
UOHI: University of Ottawa Heart Institute.
