# Supplementary figures and images for: Limitations of free-form-text diagnostic requisitions as a tool for evaluating adherence to appropriate use criteria for transthoracic echocardiography
Source: Cardiovasc Ultrasound. 2015 Jan 15;13:4. doi: 10.1186/1476-7120-13-4 (PMC4326475; doi:10.1186/1476-7120-13-4)

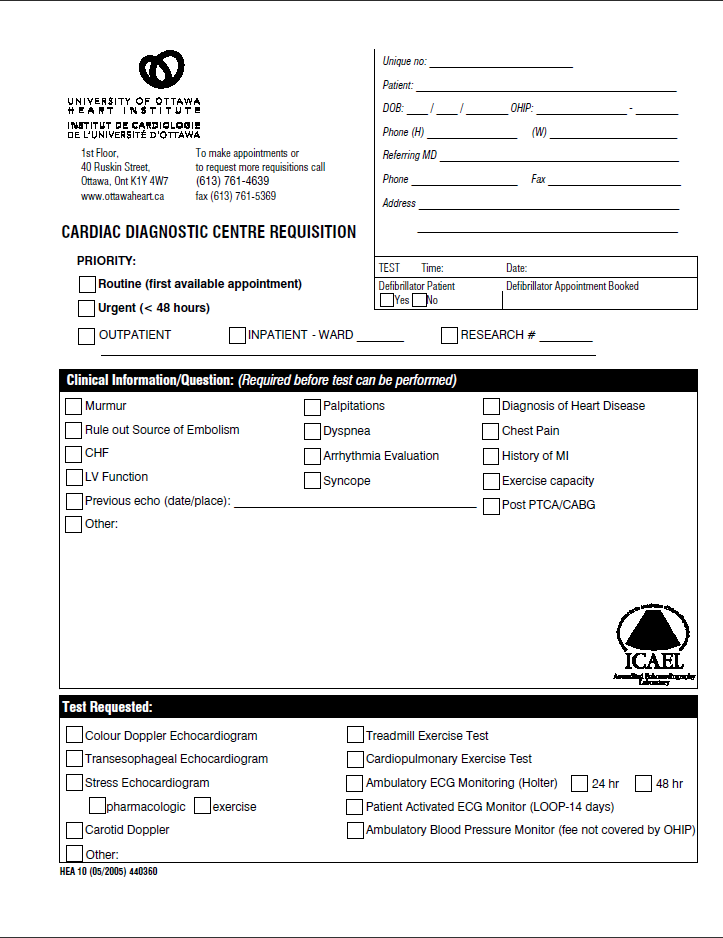

Supplement: Supplementary file 1 — Additional file 1: University of Ottawa Heart Institute Echocardiography Requisition. (PNG 95 KB) [file 12947_2014_549_MOESM1_ESM.png]
